# Annual Research Review: Psychosis in children and adolescents – a call to action: a commentary on Kelleher (2025)

**DOI:** 10.1111/jcpp.14135

**Published:** 2025-02-27

**Authors:** James G. Scott

**Affiliations:** ^1^ Child Health Research Centre The University of Queensland South Brisbane Qld Australia; ^2^ Child and Youth Mental Health Service Children's Hospital Queensland South Brisbane Qld Australia; ^3^ Queensland Centre for Mental Health Research Wacol, Brisbane Qld Australia

**Keywords:** Psychosis, children, adolescents, prevention, early intervention

## Abstract

The spectrum of psychosis is highly relevant to child and adolescent mental health. Psychotic symptoms are common in children and adolescents. The onset of psychotic disorders is often preceded by neurodevelopmental problems in early childhood, and some 13% of adolescents attending specialist mental health services will later be diagnosed with a psychotic disorder or bipolar disorder. Although 12% of psychotic disorders and 8% of schizophrenia cases have onset prior to age 18, there is little evidence available to guide the clinical care of young people with early onset psychosis. This commentary summarises the key findings of the annual research review on Psychosis in Children and Adolescents. It highlights the urgent need for clinicians and researchers in child and adolescent mental health to contribute to finding solutions to prevent the onset of psychosis and improve the lives of young people with early onset psychosis and their families.

## Background

In a herculean effort collating the available research, Kelleher (2025) provides a comprehensive review of the spectrum of psychosis in children and adolescents. Psychotic disorders were until recently considered categorical entities where onset was thought to be primarily influenced by genetic risk. Recent research utilising different methods demonstrates that psychosis is better considered a heterogeneous spectrum characterised by wide‐ranging phenotypes and variable clinical courses moderated by a multitude of genetic and environmental risk factors. Clinical precursors of psychosis are often evident years before the cardinal psychotic symptoms of impaired reality testing emerge. In addition to the fact that a substantial proportion of individuals living with psychosis experience the onset of their illness in adolescence (Solmi et al., 2022), neurodevelopmental vulnerabilities in many individuals with psychosis are present from early childhood. For this reason, child and adolescent mental health clinicians and researchers need to become more involved in the psychosis field to identify opportunities for prevention and early intervention.

## Key findings

- Psychotic symptoms are common in children and adolescents. They are often transient and have nonspecific clinical significance. While psychotic symptoms can occur in young people without mental illness, they are associated with an increased risk of a wide range of mental disorders, suicidality and functional impairment. In the absence of a psychotic illness, reassurance of young people with psychotic symptoms and their parents is indicated along with the treatment of any underlying mental health problems.
- Clinical high risk for psychosis (CHR) has attracted a great deal of attention culminating in its inclusion in DSM‐5 where the attenuated psychosis syndrome has been declared an entity for future study. However, Kelleher (2025) makes a cogent case for the limitations of CHR as a stage of illness that has clinical utility in preventing the transition to psychosis in individuals. CHR does not predict the transition to psychosis above that which occurs in young people seeking specialist mental health support; CHR services provide mental health support to a very small minority of people who will go on to develop psychosis, and clinical trials of interventions to those who meet CHR criteria have failed to demonstrate effectiveness in preventing the transition to psychosis.
- Familial high risk (FHR) of psychosis, as conferred by having one or both parents with a psychotic disorder, is also of limited clinical utility based on low sensitivity – most individuals at FHR do not go on to develop psychosis, and many who develop psychosis have no familial psychosis risk. One clinical trial of broad interventions for children at FHR has not shown any benefit at 18 months on a range of outcomes (Muller et al., 2024). Despite the allure of opportunities to prevent transition to psychosis in those who have FHR, much like CHR syndrome, there has been no meaningful clinical translation to psychosis prevention.
- Some 13% of all patients attending specialist child and adolescent psychiatry services are later diagnosed with a psychotic or bipolar disorder (Lång et al., 2022). This risk of future psychosis is comparable to individuals who meet criteria for FHR or CHR. It has been reported that neurodevelopmental disorders (ADHD, ASD) are associated with an increased risk of future psychosis; however, more recent research suggests the risk is transdiagnostic, with children and adolescents with depression, anxiety and other disorders all being associated with an increased risk of future psychosis (Lång et al., 2022). Future research on psychosis prevention necessitates studies of children and adolescents attending specialist psychiatric services.
- Although 12% of psychotic disorders and 8% of schizophrenia cases have onset prior to age 18 (Solmi et al., 2022), there is a dearth of data to inform the mental health care of early onset psychosis, which might in part account for the poor clinical outcomes of these young people. Antipsychotic therapy is effective in the acute phase of illness. As an example, clozapine is underutilised in young people and provides significant clinical benefits in individuals with early onset treatment‐refractory psychosis. However, the benefits of maintenance antipsychotic therapy are unclear. There are almost no studies of psychosocial interventions, with the available evidence suggesting that cognitive behavioural therapy and family interventions for psychosis are ineffective or possibly even harmful in early onset psychosis. Given the severe lifelong disability frequently experienced by those with early onset psychosis, there is an urgent need for further research into the clinical care of this population.

Kelleher (2025) has highlighted the paucity of evidence for clinical management and the lack of effectiveness of interventions aimed at preventing the onset of psychosis. Some opportunities to address these gaps in knowledge and practice are supported by the review by Kelleher (2025) and are as follows:

## Universal interventions are needed to reduce psychosis prevalence

McGrath et al. (2004) showed variable global incidence of schizophrenia, highlighting the possibility that psychosis incidence could be moderated by environmental factors. In terms of risk, interpersonal and social adversity such as child maltreatment, bullying victimisation, racism and discrimination increase the risk of psychosis and other serious mental disorders (Varchmin, Montag, Treusch, Kaminski, & Heinz, 2021). While high‐income countries have largely focused on providing more services to meet the rising incidence of mental illness in young people, there has been inadequate attention to public mental health initiatives that address social determinants and other risk factors for mental illness (Scott, Thomas, & Erskine, 2019).

Universal interventions that reduce childhood interpersonal trauma are critical to all mental illness prevention, including psychosis and schizophrenia. Adolescent cannabis use is another strong and modifiable risk factor. Various jurisdictions have recently legalised recreational cannabis use, with an associated increase in use by adolescents (Pawar, Firmin, Wilens, & Hammond, 2024), which might cause an increase in the prevalence of psychotic disorders in future years. Where recreational cannabis use has been legalised, measuring its use, including the preferred ways in which cannabis is consumed in adolescents who are attending specialist mental health services, would provide information on the health impact of increasing its availability in a high‐risk population for psychosis. This information is needed to inform youth‐centred cannabis harm reduction strategies. Reducing the exposure of young people to cannabis and interpersonal adversity could potentially reduce psychosis prevalence.

Public health policy must also focus on enhancing protective childhood experiences. Greater family support of children achieved through parenting skills training, nurse home visitation, access to affordable housing and childcare, as well as improved inclusivity in schools has the potential to benefit the mental health of children and adolescents.

The aetiologies for psychosis are shared with other mental disorders, and reducing their incidence is likely to have the greatest effect on psychosis prevention. In the Global North, where the incidence of mental illness is rising, mental health policy must extend beyond service provision to societal initiatives that reduce the incidence of all mental illness.

## Targeted interventions to prevent psychosis

The ineffectiveness of interventions to prevent CHR individuals from transitioning to psychosis may reflect the delivery of care in the very late stages of illness when neurobiological changes have occurred (Egerton et al., 2013). Identification of CHR and FHR may be more helpful for psychoeducation and monitoring individuals for psychosis transition rather than psychosis prevention per se.

Patients attending specialist child and adolescent services may benefit from targeted preventative interventions. These young people have a risk of future psychosis commensurate with those who meet CHR criteria; they are help‐seeking and engaged in services, but the neurobiological changes associated with their psychosis risk may be less advanced. Use of a cluster randomised trial design where safe low‐dose interventions that improve overall physical and mental health to reduce future psychosis could be trialled. In addition, linkage to electronic health records that capture future diagnoses of psychosis would enhance the feasibility of this type of study.

## Excellent mental health care for children and adolescents living with early onset psychosis

The lack of research into interventions for early onset psychosis is a serious omission in investment for child and youth mental health. Any physical disorder afflicting children and adolescents with a high probability of causing severe lifelong disability would have garnered much greater research interest. It is the responsibility of the child and adolescent mental health profession to urgently address this oversight. Young people and their families deserve to know which pharmaceutical agents are effective and tolerable in people under 18 years. Pharmaceutical industries should be incentivised to conduct clinical trials of antipsychotic agents in children and adolescents that are showing promise. Health service research examining barriers and facilitators for clozapine access in young people with treatment‐refractory psychosis is also needed.

The lack of efficacy of psychosocial interventions in early onset psychosis is surprising. Existing effective interventions in adults, such as cognitive behavioural therapy, family interventions and cognitive remediation therapy (Solmi et al., 2023) require modification so that they are developmentally appropriate and acceptable to young people and their families. They then need evaluation, and if effective, implementation. Completion of school education is critical to lifelong health and psychosocial outcomes. Children and adolescents with early onset psychosis are less likely to complete their secondary schooling, which will compromise future employment opportunities. Supporting them to complete their secondary schooling is likely to have long‐lasting health and social benefits.

## Early onset psychosis: A call to action

Kelleher's (2025) state of the art review details the available evidence of psychosis in children and adolescents and highlights the enormous gaps in knowledge of early onset psychosis prevention and management. Whilst there have been significant gains in our understanding of psychosis, there has also been a failure to make inroads into psychosis prevention, which increasingly appears to be in the purview of child and adolescent mental health. Research into the clinical care of children and adolescents with early onset psychosis has largely been neglected. It is essential that child and adolescent mental health clinicians and researchers contribute to the research effort that aims to improve the lives of the many young people living with early onset psychosis.

**Key points**

- A substantial proportion of people living with psychosis experience their onset of illness before their 18th birthday.
- There is very little evidence to inform the clinical management of early onset psychosis.
- Investment in mental health research is urgently required to prevent the onset of psychosis and improve the lives of young people living with psychosis.
